# Understanding rural fit: a qualitative study of how medical students develop rural career intentions in a socially accountable medical program

**DOI:** 10.1080/10872981.2026.2760643

**Published:** 2026-11-10

**Authors:** Wasana Jayarathne, Brendan Cantwell, Catherine Keniry, Yasmin Hawkins, Caroline Joyce, Iman Hegazi

**Affiliations:** a Rural Clinical School, Medical School, University of Queensland, Toowoomba, QLD, Australia; b School of Medicine, Western Sydney University, Penrith, NSW, Australia; c School of Rural Medicine, Charles Sturt University, Orange, NSW, Australia

**Keywords:** Rural, personal, professional, medical students, careers

## Abstract

**Background:**

Personal, educational, and contextual influences shape rural career intentions. Existing literature has focused mainly on demographic and experiential variables rather than the personal characteristics through which students experience rural practice as a meaningful vocational fit. This study explored how medical students understood the role of personal characteristics in shaping rural career intentions.

**Methods:**

We conducted an interpretive descriptive qualitative study using interviews with fourth- and final-year medical students enrolled in a joint socially accountable Australian medical program. Semi-structured interviews were analysed using reflexive thematic analysis, guided by Social Cognitive Career Theory and place attachment theory as sensitising concepts.

**Results:**

12 students participated, nine of whom had entered the program intending to practice rurally or regionally. Six interrelated themes emerged: Professional fit, Personal fit, Moral fit, Relational and place fit, Lifestyle fit, and Vulnerabilities and limits to rural fit. Across all themes, rural placements and the program's broader culture shaped students' understanding of their suitability for rural practice. These placements provided training opportunities and settings where students could assess whether rural practice aligned with their values, skills, and lifestyle preferences. Strong place attachment and community bonds played a key role, although concerns about privacy, workload, and the social dynamics of small communities also emerged.

**Conclusions:**

Participants’ rural career intentions appeared to emerge through the interaction of personal characteristics, place-based orientations and educational experiences. Rural placements functioned as developmental settings where participants tested the alignment between rural practice and their values, capacities and preferred lifestyle. Place attachment, relational orientation and informal learning through role models appeared to reinforce participants' emerging rural professional and place-based identities. As these findings reflect participants' self-described characteristics, they suggest that rural placements may help students test and consolidate their sense of fit as part of a broader bundle of rural workforce strategies.

## Background

Rural and remote communities continue to experience persistent medical workforce shortages and inequitable access to healthcare despite decades of targeted policy initiatives, including rurally located medical schools, preferential selection of rural-origin students, bonded schemes, and financial incentives [1,2]. In Australia, approximately 27−29% of the population lives in regional, rural and/or remote areas where communities experience a disproportionate burden of chronic disease, lower life expectancy and poorer access to healthcare than metropolitan populations [3]. These ongoing workforce challenges suggest that current strategies alone are insufficient and highlight the importance of understanding how medical students develop and sustain intentions to practice in rural communities.

Medical students’ career intentions are shaped by personal, educational, and contextual influences. Existing research has identified several recurring predictors of rural career intention and rural practice, particularly rural background and rural training exposure [4–6]. However, this literature has focused mainly on demographic and experiential variables, such as background, age, gender, and clinical immersion, rather than on the personal characteristics through which students come to experience rural practice as a meaningful vocational fit [7,8]. These predictors are important; however, they do not explain why some students develop a stable sense of alignment with rural practice while others with similar backgrounds and training do not.

This gap may be especially important for understanding longer-term retention. Research on rural physician retention has highlighted professional satisfaction, community belonging, work-life integration, and sense of purpose as central to continuing rural careers beyond initial recruitment [9–11]. Hence, rural career development may therefore depend not only on where students come from or what training they receive, but also on how they understand themselves in relation to rural work, rural communities, and rural places. This raises an important question: What individual characteristics shape this process of recognising and evaluating one’s fitness for rural practice?

A range of terms has been used to describe these individual-level characteristics, including non-cognitive attributes, non-academic attributes, and personal qualities [12–14]. In this study, we use the term personal characteristics, drawing on Farrington et al. [15], who describe these as a broad set of strategies, skills, attitudes, and behaviours relevant to performance and adaptation, an overarching construct as it encompasses both cognitive and non-cognitive dimensions [16]. Personal characteristics are not reducible to cognitive ability or personality traits alone. Rather, they encompass the emotional, behavioural, and interpretive orientations through which individuals engage with challenges, opportunities, and professional contexts [15]. In rural medical education, such characteristics have been proposed as relevant to practice in rural and underserved settings [17–19], although their specific form and interaction with educational experience remain insufficiently understood.

Socially accountable medical education provides a useful context for exploring these questions. Social accountability requires medical schools to align their education, research, and service activities with the priority health needs of the communities they serve [20,21]. In practice, this includes selecting students whose values and aspirations align with the school's mission and creating learning environments intended to produce graduates who are equipped and motivated to serve underserved communities.[21,22]. Alongside the formal curriculum, students are also exposed to a hidden curriculum, the informal messages conveyed through institutional culture, role modelling, and peer norms, which can strengthen or weaken their commitment to rural and underserved practice [23]. Understanding how students' personal characteristics interact with these formal and informal educational influences is therefore key to the social accountability agenda. The aim of this study is to address that gap by exploring how medical students understood the role of personal characteristics in shaping rural career intentions, including how these characteristics interacted with place-based orientations and educational experiences.

This study is supported by two theoretical perspectives. Social Cognitive Career Theory (SCCT) suggests that career interests and intentions evolve from the interplay of self-efficacy beliefs, outcome expectations, and contextual factors [24,25]. Importantly, SCCT views background factors such as personal characteristics and rural origins as influencing the learning experiences that shape these beliefs, rather than directly dictating career decisions [26]. Place attachment theory adds a dimension that SCCT does not fully capture by highlighting the affective, cognitive, and identity-based bonds individuals form with particular environments [27]. The related concept of place identity, which involves integrating the environment into one's sense of self, can help explain why some students see rural practice not just as a career choice but as a reflection of their identity [28]. Together, these frameworks provide a more comprehensive understanding of rural career development than either framework alone.

## Methodology

### Study design

This study used an interpretive descriptive qualitative design grounded in a constructivist epistemology and a relativist ontology, which assumes that meaning is co-constructed between researcher and participant and that multiple, context-dependent understandings of a phenomenon are possible and can co-exist [29–32]. This approach is frequently applied in medical education research and is well suited to exploring how personal characteristics, place-based orientations, and educational experiences interact in shaping rural career intentions. SCCT and place attachment theory served as sensitising concepts to inform interview questions and initial analysis [24,25,33]. Data were analysed using reflexive thematic analysis, which recognises the researcher's active, theoretically situated role in developing themes [34–37] which is compatible with an interpretive descriptive qualitative approach [38].

### Setting

The study was conducted within the joint 5-year medical program delivered by Western Sydney University (WSU) and Charles Sturt University (CSU). The program has an explicit social accountability mission focused on the health needs of rural and underserved communities [22,39].

### Participants and recruitment

Participants were recruited from fourth- and final-year medical students of both universities via a study flyer posted on the respective Learning Management Systems and distributed by university administrative staff. Recruitment commenced in November 2024 and continued until July 2025, when data collection was completed. The flyer included a Qualtrics link to complete a form and add interested students' email addresses. The research team then contacted interested students via email, after which they sent the participant information sheet and consent forms. Interview times were arranged after written consent was received. Fourth- and final-year students were selected because they had substantial clinical experience, had completed rural placements, and were approaching their graduation period, in which career intentions are both forming and consequential.

Twelve students participated in individual semi-structured interviews. Convenience sampling was used. All eligible students who expressed interest and provided consent during the recruitment period were interviewed. Of these, nine had entered the program intending to practice rurally or regionally, one remained undecided, and two described a change in intention following rural placements, one towards and one away from rural practice. This variation was sought to explore both stable and changing rural career intentions. We considered the adequacy of the sample using the qualitative concept of information power [40], consistent with the assumptions of reflexive thematic analysis [34–36]. The study’s focused aim, the specific sample, interview richness, and analytical approach indicated that 12 interviews provided sufficient information power for the analysis.

### Data collection

The first author conducted all interviews via Zoom. Interviews lasted approximately 30 to 45 minutes. All interviews were audio-recorded with written informed consent and transcribed verbatim by a professional transcription service. The interview guide was developed collaboratively by the research team, using SCCT and place attachment theory as sensitising lenses [33]. Interviews began with questions about participants' backgrounds, including whether they had grown up in rural or urban settings, and then explored motivations for studying medicine and their intended career direction upon entering the program. Participants were then asked how their rural career intentions had developed or changed over time and which experiences had shaped those changes. Questions also invited direct reflection on personal characteristics, including how these might support or hinder rural practice. Additional prompts explored the influence of placements and curriculum content.

### Data analysis

Analysis was led by the first author, who coded all 12 transcripts, attending to both semantic and latent meanings. Coding was primarily inductive and grounded in the data, with SCCT and place attachment theory serving as relevant sensitising concepts. Another team member independently coded all transcripts, bringing a second interpretive perspective to the data. Rather than reconciling codes or seeking agreement, two researchers compared interpretations, discussed areas of convergence and divergence, and questioned the assumptions behind codes. This was not undertaken to establish inter-rater reliability, which is inconsistent with the epistemological assumptions of reflexive thematic analysis [34,35]. Rather, it served as an analytic resource, providing additional interpretive reading and helping surface assumptions that might otherwise have remained implicit [34,35]. The first author then actively developed the themes, clustering patterns of shared meanings organised around the central concept of “participant's sense of fit with rural practice. Then these themes and subthemes were reviewed against the full dataset, and refined iteratively through writing process and discussion with the wider research team. Trustworthiness was supported through sustained engagement with the dataset, interpretive dialogue between researchers, and transparent documentation of the analytic process [41].

### Ethical clearance

This study was conducted in accordance with the ethical principles of the Declaration of Helsinki and its subsequent amendments. Ethical approval was obtained from the Human Research Ethics Committees at (both Western Sydney University (WSU) and Charles Sturt University (CSU), Australia (H16220 and H24362, respectively) before data collection commenced. Participants were provided with detailed information about the study before participation (including the study team's contact information in case they needed to ask questions) and gave written informed consent. The study was classified as low-risk research, and all data were collected, stored, and analysed in accordance with approved ethical procedures and the ethics committees at WSU and CSU, Australia.

### Reflexivity

Our research team held academic and clinical roles within the joint program under study. This positionality shaped interview guide preparation, participant interaction, interpretation, and representation of the findings. To address this, the research team held regular discussions to examine and challenge interpretive positions. Consistent with reflexive thematic analysis, the analysis is therefore presented not as a neutral account of participant experience, but as a theoretically informed interpretation [34,35].

## Results

### Participant characteristics

Twelve students participated. Fourth year and final-year students; 10 female and 2 male who nine of them described rural and regional upbringing (Table 1). Nine participants entered the programme intending to practice rurally or regionally; two changed their intention after rural placements, one towards and one away from rural practice. All participants had completed short-term/long-term rural placements before their interviews.

**Table 1.** Participant Characteristics.

Participant	Year of study	Gender	Rural/regional background	Rural placement exposure	Rural/regional Career intentions at entry
P10	Year 4	Male	Yes	Yes	Yes
P11	Year 4	Female	Yes	Yes	Yes
P12	Year 5 (Final year)	Female	No	Yes	No decision at entry and long-term need to work in rurally
P13	Year 4	Female	No	Yes	Yes
P14	Year 4	Female	Yes	Yes	Yes
P15	Year 4	Male	Yes	Yes	Yes
P16	Year 4	Female	Yes	Yes	Yes
P17	Year 4	Female	Yes	Yes	Yes
P18	Year 4	Female	No	Yes	No, and later changed towards rural
P19	Year 4	Female	Yes	Yes	Yes
P20	Year 5 (Final year)	Female	Yes	Yes	Not decided at entry and remain undecided
P21	Year 4	Female	Yes	Yes	Yes, Intentions changed towards urban

Six interrelated themes were identified relating to the personal characteristics associated with rural career intentions: 1. Professional fit 2. Personal fit 3. Moral fit 4. Relational and place fit 5. Lifestyle fit 6. Vulnerabilities and limits to rural fit. Across all themes, participants described rural placements and the program's broader culture as shaping their understanding of their suitability for rural practice. All subthemes and themes are summarised in Figure 1 and Table 2.

**Figure 1.**
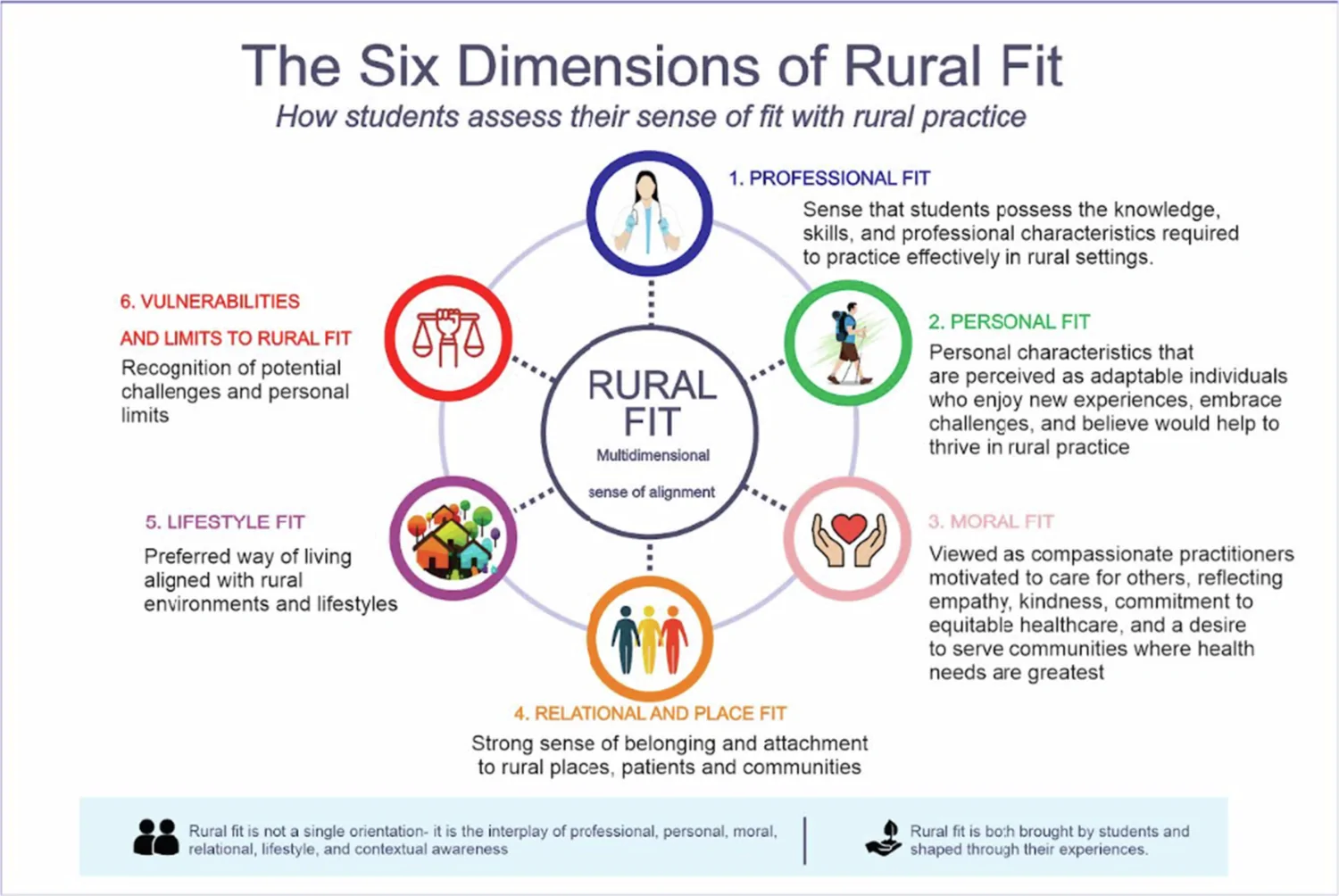
Six dimensions of rural fit.

### Theme 1: professional fit

The first theme captured participants' sense that they possessed the knowledge, skills, and professional characteristics required to practice effectively in rural settings.

a. ***Self-driven***

Students commonly described themselves as self-motivated, optimistic, accountable, and capable of solving problems independently. These qualities were presented as established aspects of self rather than as characteristics they hoped to develop.

*Note: Participants' characteristics accompanying quotations are presented as follows: Yr = Year of study, F = Female, and M = Male.*

*“I think it's good that I come from the country. Obviously I don't think that is an exclusive quality, but I think it is good that I come from the country. I'm very community minded and very active in community building and engagement as well….I’m an energetic person and I'm an optimistic person*. *I think the energy and bubbliness that I bring to the clinical setting, as well, is a skill in itself and being responsible for team morale a little bit.* *”* (P16, Yr4, F)

b. ***Accountable***

Participants often linked these characteristics to the demands of rural work, describing rural practice as suited to people who were comfortable taking initiative and responsibility.

*“That's a big skill of mine is organising teams and helping people to take ownership of projects and decisions and responsibility to get the team to work at its best.”* (P10, Yr4, M)

Within this subtheme, participants regarded taking ownership of team decisions as well suited to rural practice, where, as noted above, they expected to take initiative and assume responsibility.

c. ***Independent and considered decision-maker***

Participants spoke of learning that they could function in unfamiliar settings, respond to changing demands, and manage broader clinical expectations. Participants described being drawn to settings in which they would need to think broadly and continue developing new skills.

*“You have limited resources. You need to be able to be - like work independently. I think all the other things, you need to be reliable, adaptable, flexible.* *”* (P11, Yr4, F)

### Theme 2: personal fit

Students perceived themselves as adaptable individuals who enjoy new experiences, embrace challenges, and take initiative. Students described personal characteristics they believed would help them thrive in rural practice and meet its unique demands.

a. ***Adaptability and flexibility***

Participants described flexibility and adaptability as important qualities for practicing in rural settings. Rural placements were often described as experiences that confirmed these perceptions.

*“Yeah, I think maybe like flexibility, a willingness to learn new things and get exposure to a variety of settings. I feel like I'm quite adaptable to different environments as well, yeah.”* (P 11, Yr4, F)

b. ***Novelty-seeking and risk-taking***

Some identified themselves as people who actively sought variety and were comfortable with risk. These characteristics shaped their view of rural practice, which was often framed as interesting, dynamic, and professionally stimulating. For these participants, the breadth and unpredictability of rural work were experienced as attractive rather than discouraging.

*“love novelty. I absolutely seek out novelty.”* (P16, Yr4, F)

*“I think something I'm worried about in career choice is picking something that will make me bored eventually, and having such a broad scope is something that would be good for me to be able to be able to do different things every single day and really have variety in the workplace.”* (PI2, Yr5, F)

c. ***Curiosity and challenge orientation***

Related to novelty-seeking was a strong orientation toward curiosity and challenge.

Several participants linked this orientation to what they had observed during rural placements, where rural clinicians were seen as managing a wide scope of practice and engaging in varied, meaningful work.

*“I'm quite happy to do things that I haven't done before and try new things and stuff like that, which I think really helps, at least as a medical student over here, but also as a doctor later on, when you see stuff and it's an emergency and you just need to be confident and just do it and give it a go, which I think I'm quite happy to do that.”* (P18, Yr4, F)

### Theme 3: moral fit–

Students viewed themselves as compassionate practitioners motivated to care for others. Their descriptions reflect empathy, kindness, commitment to equitable healthcare, and a desire to serve communities where health needs are greatest.

a. ***Empathy***

Participants also described empathy as a personal characteristic that aligned well with rural and underserved practice. These accounts positioned empathy as both part of how participants understood themselves and part of what drew them to rural work.

*“A bit of empathy, yeah, but then also I think, understanding of how difficult it can be to get an appointment in a rural area and get things done, get the help you need. I think it means - yeah, since I had that understanding of how difficult it can be, it makes me want to work a bit harder and help patients get what they need. Yeah”.* (PI4, Yr4, F)

b. ***Social justice and service orientation***

A further subtheme concerned equity, service, and community need. Several participants linked their rural intentions to personal or family experience of limited healthcare access, or to a broader commitment to underserved communities. In these accounts, rural practice was described not only as personally appealing, but also as socially meaningful.

*“I was doing Aboriginal studies, and I found out that the young people in the community were getting Type II diabetes as kids, so I thought that was a really big issue and thought, by becoming a doctor, maybe I’d be able to help close the gap.”* (P19, Yr4, F)

*“Well, in the town of (name of large rural town in Country redacted) we only had a locum doctor in the ED, so sometimes if you wanted care it would depend on that locum doctor being there and then sometimes, they'd say, there's not going to be a doctor tomorrow so you'll have to drive to the next nearest town, which is two hours away, so that was really hard growing up.”* (P19, Yr4, F)

### Theme 4: relational and place fit

The fourth theme captured the relational values participants attached to rural practice. Students perceived a strong sense of belonging and attachment to rural places and communities. They valued close interpersonal relationships, community engagement and familiarity with patients.

a. ***Community-minded***

Many portrayed themselves as community-minded and connection-oriented, and viewed rural settings as more compatible with these qualities than metropolitan environments. They valued the prospect of getting to know patients, families, and communities over time. Continuity of care and the possibility of becoming part of a community were recurring features of these accounts.

*“Yeah, so I find whenever I’m doing placement in semi-rural areas that there’s just more family orientated, or they have more connection with the patients. I actually value that as well, so that really aligns with what I like and what—that sense of community, that sense of, we’re treating everyone in our community and stuff.”* (P 21, Yr4, F)

b. ***Attached to rural people***

Participants described emotional connections with patients, families and community members as a source of interpersonal fulfilment. They perceived that a close-knit rural environment enabled doctors to build deeper, more personal relationships with patients, allowing them to spend time listening, engaging, and sharing in patients’ experiences. Such connections contributed to greater professional satisfaction and reinforced participants’ attachment to rural practice and rural communities.

*“think, for me, I really enjoy talking to patients and being a part of their life. I think that's something that people really, really want from their doctors, and I think that really works in a rural hospital, because you have that time to talk to them and laugh with them and spend time with them and make sure that they have a good experience when they are rural.* *”* (P18, Yr4, F)

c. ***Sense of belonging***

Some participants described feeling accepted and socially embedded in the rural communities where they undertook placements.

*“I think you get to really connect with the community here, make good friendships. You're not far away from anyone as well. You get to meet people more often. You see everyone on the street and stuff. It's nice.* *”* (P18, Yr4, F)

### Theme 5: lifestyle fit

Students believed that their preferred way of living aligned with rural environments and lifestyles.

a. ***Pace of life and lifestyle alignment***

Students viewed rural settings as offering a pace and quality of life that aligned with how they wanted to live and work. For many, this alignment was tied to the perceived sustainability of a future rural career.

*“The pace of life, the community feel, all of those things fit into what I enjoy and what I want to build in my life I guess.”* (P16, Yr4, F)

*“Perhaps the speed here is a little slower. We don't have the extreme time pressure or the extreme bed pressure that metro hospitals have to quickly discharge a patient or to get them out fast.”* (P13, Yr4, F)

b. ***Dislike of urban environments***

Participants not only expressed attraction to rural settings, but some also described urban environments as actively unattractive. Cities were portrayed as crowded, impersonal, or competitive, and as less compatible with participants' values and preferred way of life.

These accounts sharpened the contrast between rural and urban practice and reinforced participants' sense of rural fit.

*“I think I just got sick of cities (P10, Yr4, M) the city is bustling it's hard to get around as well. I feel like, logistically, transport, driving is a nightmare.”* (P17, Yr4, F)

*“Yeah, my plan was always to stay at least regional-rural, and if I’m living rurally, I wouldn’t mind working remotely, potentially, depending on where my career ends up, but yeah, I never planned to go to the city.”* (P17, Yr4, F)

c. ***Connection to natural environments***

Participants also embodied a sense of comfort in rural and natural environments. They referred to open spaces, agricultural landscapes, and a general feeling of being more at ease outside metropolitan settings. For some, this connection appeared longstanding; for others, it became clear through rural placements and spending time in rural communities.

*“Then you talk to these people, and they’re like, yeah, I’m gonna feed my horses and I’m teaching my kids to do this, and really involved in their kids’ lives, really involved in their actual life, and not just work, work, work, work, which I thought was really nice. That’s what I would like to do in the future. Have a few horses, enjoy my life. So I think that’s what I liked.”* (P18, Yr4)

*“I like the idea that I can have land, a bit of space, a nice big house, and not have neighbours really close by. Things like that, which are all things that I wouldn't be able to get living in the city.”* (P14, Yr4, F)

### Theme 6: vulnerabilities and limits to rural fit

The last theme captured factors that complicated participants’ sense of fitness with rural practice.

a. ***Privacy and blurred boundaries***

One commonly identified issue was reduced privacy in small communities. The same closeness that participants valued was also seen as creating tension between personal and professional life. Participants differed in how strongly they experienced this as a concern, but it was recognised as a real feature of rural practice.

*“I do like to separate my private life. I do like to have a bit of privacy. My experience has been, say, working in very small rural towns, I think everyone in the community knows everyone’s business.”* (P15, Yr4, M)

b. ***Work-life balance and professional isolation***

Some participants were also concerned about workload, professional isolation, and the demands of small-community practice. They described rural work as meaningful but potentially difficult to sustain. These concerns were often expressed alongside positive rural intentions, indicating that participants' accounts were not idealised.

*“Yeah. I think in rural areas, the work-life balance isn't as good. That makes it difficult.* *”*(P14, Yr4, F)

*“I'm getting a bit nervous about, what if I'm not really sure what I'm doing and I won't have that additional support. If I'm in the city. I've got 10 people I can call. But out rural, sometimes it might just be me, and I do - that does make me worry, what if I don't know what I'm doing in a situation, and I don't have someone to call. I think that's one thing that might make it a bit more difficult to work in a rural area, not having that support.”* (P14, Yr4, F)

**Table 2.** Themes and subthemes.

Themes	Subthemes
Professional fit	*Self-driven* *Accountable* *Independent and considered decision maker*
Personal fit	*Adaptability and flexibility* *Novelty-seeking and risk-taking* *Curiosity and challenge orientation*
Moral fit	*Empathy* *Social justice and service orientation*
Relational and place fit	*Community-minded* *Attached to rural people* *Sense of belonging*
Lifestyle fit	*Pace of life and lifestyle alignment* *Dislike of urban environments* *Connection to natural environments*
Vulnerabilities and limits to rural fit	*Privacy and blurred boundaries* *Work-life balance and professional isolation*

## Discussion

Our study explored how medical students in a socially accountable program understood the role of personal characteristics in shaping their sense of fit with rural practice and how these characteristics were experienced in relation to rural places and educational experiences. Rural career intentions were described not simply as effects of background or exposure, but as developing through an interaction between self-understanding, experience of rural places and training. Three findings are particularly relevant and significant for rural medical education and workforce planning, recognising that the study examined perceived fit and career intentions rather than actual career choices or retention.

### Rural placements as context for career fit

Participants did not describe personal characteristics as fixed characteristics that independently determine their career intentions. Instead, students described rural placements as providing opportunities to explore, test, and clarify preexisting orientations towards rural practice [42,43]. These experiences appeared less important for creating new personal characteristics than for helping students recognise whether rural practice fit with how they already understood themselves. Thus, rural immersion appeared to function as a form of career clarification, enabling students to evaluate their compatibility with rural medical practice. Kristof Brown et al. [44] define in-person job fit as the alignment between an individual and their job. Participants' accounts suggest that perceived person-job fit may contribute to their interest in and openness to rural practice. Additionally, our findings reveal that participants also appear to evaluate whether their personal identity matches their preferred practice style and the professional and lifestyle environment they aspire to.

This interpretation is consistent with SCCT, in which learning experiences shape the self-efficacy beliefs and outcome expectations that inform career intentions [26]. Additionally, rural self-efficacy provides a useful lens for understanding these findings, as participants described experiences that built confidence in their ability to practice in rural settings [45]. It also aligns with professional identity formation theories, which discuss how identity is developed through socialisation, reflection and experience rather than being fixed at entry to medical school [46,47]. The characteristics participants attributed to themselves formed a coherent pattern compatible with rural practice, including self-drive, adaptability, and confidence in working with limited resources, a desire for variety, novelty, broad responsibility, strong relational and community-minded orientations, and values centred on service and social justice, similar to previous research [16].

These findings may have implications for rural medical education, although they should be interpreted cautiously. Rural workforce strategies often treat selection and training as separate interventions [48], with efforts predominantly focused on recruiting students from rural backgrounds while increasing exposure to rural clinical placements during medical training [43,48,49]. Our findings suggest that a more dynamic relationship exists between these processes, as most participants entered the program already intending to practice rurally. Although selection may identify students with characteristics and interests that predispose them towards rural practice [42,43,50], educational experiences play a critical role in confirming, strengthening, and challenging that alignment highlighting the role of rural placements in shaping rural intentions. Thus, students' experiences suggest that rural career intentions are influenced by multiple interacting factors rather than any single intervention. This reinforces the importance of integrated rural workforce pathways that combine education, support and retention strategies.

### Role modelling, informal learning and social accountability

Participants' accounts referred to learning that occurred alongside the formal curriculum; participants described observing rural clinicians managing a broad scope of practice and engaging in varied meaningful work (Themes 2 C), valuing the community orientation and continuity of relationships they encountered on placements (Themes 4a) and perceiving the less pressured working culture in rural hospitals (Theme 5a). We interpret these accounts through the lens of hidden curriculum, informal messages conveyed through culture, role modelling and peer norms [23], which may contribute to how students see rural practice as feasible and desirable [43,51,52]. Rural placements send implicit messages about practice, valued identities, and meaningful work, aligning with the view of socialisation [23,53,54]. Our findings suggest the informal learning mainly reinforces the program's mission, with students observing engaged, fulfilled rural clinicians, which helps them see rural practice as feasible and desirable. Visible examples of clinicians whose work values and lifestyle align with rural practice will help students to develop their career goals and professional identities [55]. This alignment can strengthen the link between institutional goals, student values, and clinical experience, consistent with the recent curriculum mapping research showing that competencies such as culturally responsive care benefit from a longitudinal, interconnected curriculum where foundational knowledge is progressively reinforced and applied clinically [56]. For program design, informal messages and role modelling should be part of the educational approach, not secondary, emphasising supervisor selection and how rural work is discussed alongside formal curriculum content.

### Place attachment in rural career development

Place attachment is prominent in participants' narratives. Students described rural career intentions not only in terms of work content, but also through attachment to the rural environment. For some, this was an attraction to rural pace, space, and community life; for others, it was reinforced by dislike of urban environments. These findings suggest that place-based orientation may be more than a background feature of rural career choice; it may form part of the mechanism through which students come to see rural practice as desirable and sustainable [43,49,57]. This suggests an emerging or developing place identity, which might clarify why rural intention is often experienced not just as a career choice but as a feeling of personal and professional connection [28]. However, some students' views on rural fit were mixed: while attracted to rural pace, space, and community, some expressed concerns about practice demands, costs, loss of privacy, work-life balance, and the risks of being a single doctor in a small town [49,58]. Their honest doubts highlight practical and relational challenges they see as important for a sustainable rural career. Consequently, rural placement design may benefit from promoting continuity of community engagement, opportunities for participation in local life, and structured reflection on place, belonging, and professional identity [56].

These findings have implications for socially accountable medical education, highlighting the value of using rural fit as a framework for structured reflection, designing placements that build rural self-efficacy through authentic clinical and community engagement, and explicitly addressing the challenges of rural practice through mentoring and education. Nonetheless, whether these approaches ultimately improve workforce outcomes and retention requires longitudinal evaluation.

### Limitations

As with all qualitative research, our findings should be considered within their limitations. Our study involved 12 students from two medical schools with a socially accountable mission. Thus, findings are contextually situated, and their transferability to programs with different missions, selection processes or placements remains to be tested. Second, nine of the 12 participants had entered the programme already intending to practice rurally or regionally. Thus, participants may have been more interested in rural practice than students who did not participate. This may have heightened the emphasis on rural commitment, place attachment, and relational values within the data. Additionally, the study captured self-reported intentions and interpretations at a single point in time. This cannot determine whether those intentions translate into future practice or long-term retention. Longitudinal work following students into postgraduate training and practice would strengthen understanding of how personal characteristics, educational experiences and place attachment interact over time.

## Conclusions

Participants' rural career intentions appeared to be shaped by a dynamic interaction among personal characteristics, place-based orientations and educational experiences. Participants described rural placements not simply as training sites or opportunities for exposure, but as settings in which they tested and clarified whether rural practice suited their values, capacities and preferred way of life. Their self-described characteristics appeared as an emerging rural orientation in the process of forming rather than as a fixed set of characteristics. Place attachment and, relationships and rural role models appeared to reinforce developing rural professional identity. Together, these findings position rural placements as developmental sites where students actively shape career alignments and can test and strengthen their sense of rural fit, supporting rural careers within broader workforce strategies.

## Supplementary Material

Reflexive Thematic Analysis Reporting Guidelines.docx Reflexive Thematic Analysis Reporting Guidelines.docx

## Data Availability

The data that support the findings of this study are available from the corresponding author upon reasonable request. Access to the data may be subject to ethical and institutional requirements to protect participant confidentiality and privacy.
