# Supplementary material for: Understanding rural fit: a qualitative study of how medical students develop rural career intentions in a socially accountable medical program
Source: Med Educ Online. 2026 Nov 10;31(1):2760643. doi: 10.1080/10872981.2026.2760643 (PMC13757828; doi:10.1080/10872981.2026.2760643)
Supplement: Supplementary file 1 [file ZMEO_A_2760643_SM7877.docx]

**Reflexive Thematic Analysis Reporting Guidelines (RTARG) (Braun & Clarke, 2024)**

Braun V, Clarke V. Supporting best practice in reflexive thematic analysis reporting in Palliative Medicine: A review of published research and introduction to the Reflexive Thematic Analysis Reporting Guidelines (RTARG). Palliative medicine. 2024 Jun;38(6):608-16.

| **Advice for aspects of the research report/approach to reporting** | | **Line numbers** |
| --- | --- | --- |
| The Introduction  NB: We prefer Introduction over Literature Review as a section heading, to capture the broader purpose of this section. | Provide a robust context and rationale for the proposed research in the Introduction. | 45-111 |
|  | Clearly articulate a research question – one that is methodologically coherent. | 45-111 |
|  | Include information on guiding theoretical assumptions and other (e.g., explanatory)theory informing the use of TA. | 101-111 |
|  | Report in a way that is consistent with stated theoretical assumptions throughout. | Philosophical assumptions and methodological assumptions of reflexive thematic analysis are maintained throughout the study design, analysis, interpretations and reporting  In full manuscript |
|  | Evidence methodological coherence/integrity in both the research and the report.7 |  |
|  | Show evidence of reflexive practice and Write in a methodologically coherent style. | 190-196 |
| The Methodology  NB: We prefer the theoretically-embedded term Methodology as a section header, over the proceduralist term Method. | Describe selection of participants/data items. | 136-164 |
|  | Describe number of participants/data items; provide a rationale or explanation around dataset or participant group size/composition. | 166-178 |
|  | Discuss characteristics of participants/data items. | 136-150 |
|  | Detail ethical approval and ethical code/principles followed, participant informed consent, etc. | 180-188 |
| Dataset generation  NB: We prefer the term generation over collection to capture the active role of the researcher and that data don’t pre-exist research as data, but become data through research practices | Provide some rationale for method(s) for data generation/data item sources chosen. | 114-126 |
|  | Describe development and/or characteristics of data generation tool(s). | 136-164 |
|  | Include details such as modality and/or setting of data generation, time frame, and other pertinent procedural information. | 137-164 |
|  | Describe who conducted any interactive data generation (which author or research role), and how. | 154-164 |
|  | Describe the size/scope of the dataset and dataset items. Describe, and if relevant explain, any preparation of data for analysis. | 167-178 |
|  | Provide some rationale for use of RTA, and, where relevant, for combining RTA with other approaches and procedures. | 170-178 |
|  | Describe specific orientation to RTA. | 116-126 |
|  | Discuss how the researcher(s) engaged with the analytic process. | 167-178  190-195 |
|  | Where more than one person is involved, describe who analysed the data (author or research role). | 167-178 |
|  | Use language to describe the process and products of RTA that is coherent with the values and assumptions of RTA. | 116-126 |
| The Analysis  NB: We prefer the heading Analysis over Findings/Results. Findings implies the researcher “found”, “discovered” or “identified” pre-existing themes. Results is strongly associated with the outputs of statistical analysis. | Provide an overview of themes or thematic structure | 198-209 |
|  | Ensure theme conceptualisation is appropriate to RTA, and any divergences are justified and explained. | 198-209 |
|  | Name themes appropriately. Report themes in sufficient depth and detail. Use subthemes judiciously. | 198-429 |
|  | Ensure the analytic narrative explains the meaning and significance of the data. Provide an appropriate balance of analytic narrative and data extracts – both data extracts and analytic narrative matter. |  |
|  | Demonstrate coherence between analytic narrative and illustrative/evidentiary data extracts. Integrate existing research and theory into the analytic narrative. |  |
| The Final Section – A General Discussion or “Conclusions”  NB: We don’t have a preference for what a final section of an RTA report is called, and it depends on the context and the focus and purpose of the study – the heading Conclusion may evoke a certainty that isn’t appropriate; Implications may be useful; Final Considerations or Reflections may work, as might General Discussion. | Draw analytic conclusions across themes. | 430-532 |
|  | Discuss implications or directions for future research | 430-504 |
|  | Use and report quality practices coherent with RTA. | A detailed description of researcher reflexivity, analytical procedure, theme development, and theoretical positioning is reported. Transparency was further supported through the completion of the reporting guidelines and inclusion of line numbers for peer review. |
|  | Evaluate the research from a Big Q standpoint | Research quality was appraised through methodological coherence, reflexivity, transparency, interpretative depth and contribution to understanding consistent with Big Q qualitative research |
